# White Blood Cells, Platelets, Red Blood Cells and Gas Bubbles in SCUBA Diving: Is There a Relationship?

**DOI:** 10.3390/healthcare10020182

**Published:** 2022-01-18

**Authors:** Danilo Cialoni, Andrea Brizzolari, Alessandra Barassi, Gerardo Bosco, Massimo Pieri, Valentina Lancellotti, Alessandro Marroni

**Affiliations:** 1 Environmental Physiology and Medicine Laboratory, Department of Biomedical Sciences, University of Padova, 35100 Padova, Italy; andreabrizzolari79@gmail.com (A.B.); gerardo.bosco@unipd.it (G.B.); 2 DAN Europe Research Division, 64026 Roseto degli Abruzzi, Italy; mpieri@daneurope.org (M.P.); amarroni@daneurope.org (A.M.); 3 Department of Health Sciences, Università degli Studi di Milano, 20142 Milan, Italy; alessandra.barassi@unimi.it; 4 Cardiothoracic and Vascular Department, Azienda Ospedaliero Universitaria Pisana (AOUP), 56100 Pisa, Italy; vallylancellotti@gmail.com

**Keywords:** SCUBA diving, diving, hyperbaric medicine, white blood cells, platelets, red blood cells, inert gas embolism

## Abstract

(1) Background: SCUBA diving can influence changes of several hematological parameters (HP) but the changes of HP in the decompression phases are still unclear. The aim of this study was to investigate any possible relationship between HP and predisposition to inert gas bubble formation after a single recreational dive. (2) Methods: Blood, obtained from 32 expert SCUBA divers, was tested for differences in white blood cells (WBC), granulocytes (GRAN), lymphocytes (LYM), and monocytes (MONO), red blood cells (RBC), and platelets (PLT) between bubblers (B) and non-bubblers (NB). (3) Results: We found inter-subject differences in bubble formation (considering the same diving profile performed by the divers) and a statistically significant higher number of total WBC, GRAN and LYM in NB as compared to the B divers in the pre and in the post diving sample, while no statistical differences were found for MONO and PLT. In addition, we did not find any statistically significant difference between NB and B in RBC. (4) Conclusions: Our results, even if in absence of investigated anti-inflammatory markers, could indicate a relationship between low WBC numbers and bubble formation. This aspect may explain a possible cause of inter-subject differences in bubble formation in divers performing the same dive profile.

## 1. Introduction

Self-contained underwater breathing apparatus (SCUBA) diving exposes the human body to changes in environmental conditions including hyperbaric exposure, breathing compressed air (or other gas mixtures) at elevated pressure, and often exposure to cold temperature. In addition, diving involves physical activity due to the increased breathing and movement resistance and effort to manage the diving equipment [1,2].

Type of dive (recreational or professional), depth, diving time and associated physical exercise can influence the hemodynamic response in divers and all these variables can originate physical and psychological stress inducing several body adaptations [3,4]. Among the many parameters that show changes during and after diving, hematological parameters and particularly white blood cells (WBC) and platelets (PLT) changes are particularly interesting [5].

Some authors studied male divers after a single recreational dive at 30 m for 30 min [5], and they found a rapid increase of neutrophils, the most abundant type of granulocytes (GRAN), after surfacing and of lymphocytes (LYM) after 6 h. On the other hand, monocyte (MONO) count decreased immediately after surfacing to increase again 6 h after diving [5]. The neutrophil mobilization may be inducted by the stress associated to the dive that results in an increased release of cortisol and catecholamines [6,7,8] and also influenced by acute exercise, resulting in a rapid accumulation of neutrophils in muscles, related to both intensity and duration of exercise [9]. Neutrophils may be activated during exercise by several factors including muscle damage, growth hormone and IL-6 [10,11]. Activated neutrophils could phagocytate cellular debris and release growth factors that recruit macrophages, which are involved in removing residual cell fragments and in reconstructing muscle fiber [12]. In addition, nitric oxide (NO), frequently studied in SCUBA diving for its role in the arterial flow regulation, exerts an important action on various neutrophil cells activities. NO plays a protective role in neutrophil-endothelial interaction by preventing neutrophil adhesion and endothelial cell damage by activated neutrophils [13]. Furthermore cold exposure during diving might influence WBC mobilization: some authors investigated haematological changes during a long swimming competition in cold water (6 °C) [14]. LYM increase H_2_O_2_ production after scuba diving, in normobaric condition, probably as a consequence of some mitochondrial changes [15]. After a hyperbaric exposure (e.g., scuba diving) LYM activate their antioxidant defenses in order to protect the cells against the induction of oxidative damage of macromolecules, especially DNA [16,17]. Increased levels of reactive oxygen species (ROS) can activate the antioxidant machinery of LYM, and GPx is one of the first antioxidant systems activated to detoxify ROS [15]. According to these data, the increase of LYM observed by Perovic et al. may be a consequence of the combination of physical activity, physiological stress, hyperbaria, hyperoxia and exposure to cold [5]. Physical exercise can induce a mild monocytosis often accompanied by neutrophil increase in the inflammatory response [18], especially in cold water [14]: it has been observed that MONO decreased immediately after diving [5]. MONO reduction may be due to a trans-endothelial migration caused by the alterations that can occur in the vascular/endothelial function observed after dive [19,20].

Red blood cells (RBC) are exposed to ROS and reactive nitrogen species (RNS) attack because of the high polyunsaturated free fatty acid content of their membranes and the high cellular concentrations of hemoglobin [21] but contain an elaborate endogenous antioxidant defense system that eliminates free radicals [22]. The oxidative membrane damage can be the reason of the RBC reduction observed in SCUBA divers by some researchers [5]: this may be due to increased pO_2_ resulting from hyperbaric exposure during diving that could induce oxidative stress by raising ROS generation [23].

It is well-known that SCUBA diving can increase the number of vascular gas emboli (VGE), that may further exacerbate the endothelial homeostasis already impaired by ischaemia/reperfusion, physical contact or by an increased shear stress [19]. Several studies have focused on diving-related VGE formation [24,25,26] that plays a key role on the onset of decompression sickness (DCS). Notwithstanding the frequent presence of “silent” asymptomatic VGE in divers after diving, the link between circulating VGE and DCS is well accepted [27,28].

PLT may have a key role in the onset of DCS [29]. Circulating bubbles can alter the clotting system both through activation of the coagulation cascade and the induction of PLT aggregation while circulating proteins adhere on bubble surface generating a thin layer that interacts with PLT [30]. PLT are one of the main sources of circulating microparticles (MP) [31]. During PLT activation, PLT-derived MP are generated in the bloodstream: some authors found a relationship between post-dive decrease of PLT-derived MP concentration reflecting PLT activation and bubble formation, probably due to their pro-coagulant activity that leads to the alteration of coagulation and thrombotic events in the pathogenesis of DCS [32].

The aim of this study was to investigate for possible relationship between hematological parameters and the predisposition to inert gas bubble formation after SCUBA diving.

## 2. Materials and Methods

### 2.1. Subjects and Diving Procedures

A total of 32 expert SCUBA divers (26 males and 6 females) were investigated during a single ricreational dive in Y-40 “The Deep Joy” swimming pool (Montegrotto Terme, PD), 42 m depth.

No subject reported previous episodes of DCS, historical or clinical evidence of arterial hypertension, cardiac, pulmonary or any other significant disease, none of them took prescription drugs, suffered any acute disease during the 15 days before the experiment, or reported assumption of anti-inflammatory drugs and exposure to high altitude in the 7 days before the experiment. All the divers received an explanation of the study’s purposes, risks and benefits, were familiarized with the experimental protocol and read and signed a specific informed consent form before the experiment. The study was conducted in accordance with the Helsinki Declaration and was approved by the Ethical Committee of Università degli Studi di Milano, Italy (Aut. n° 37/17). No diver performed any compressed-gas diving or any breath- hold diving during 30 days before the day of the experiment.

Divers were invitated to reach a bottom time of 7 min (descent and permanence at the bottom) so as not to exceed the no-decompretion limit. After the dive, all the diving profile were downloaded into the divers alert network (DAN) database using a common export fomat called universal dive data format (UDDF) and the gradient factor (GF) was calculated by a dedicate DAN Software using the Buhlmann ZHL16 C model and used to ascertain uniformity of hyperbaric exposure in addition to the analysis of compliance with the suggested diving profile.

The maximum GF value is generally reached at the end of the dive. The GF measures the inert gas load in the diver’s tissues, according to the selected decompression algorithm. This is a way to estimate inert gas supersaturation and to compare diving exposure in the different investigated subjects [25].

### 2.2. Material and Protocol

Venous blood samples were obtained from the antecubital vein of divers in heparin containing tubes (Vacutainer, Becton, Dickinson and Company, Franklin Lakes, NJ, USA). Blood samples were collected 30 min before the diving session and 30 min after surfacing (post diving).

All haematological analyses were performed within an hour after blood sampling on the same Abaxis Piccolo Xpress^®^ chemistry analyzer (Union City, CA, USA). Briefly, 100 µL of whole blood were transferred into the self-contained reagent disc. The disposable, single-use disc contains all the reagents and the diluent necessary to perform a complete multi-test chemistry panel. The following haematological parameters were measured: WBC, WBC differential blood count including, GRAN, LYM, and MONO, RBC, haemoglobin (HGB), mean corpuscular volume (MCV), haematocrit (HCT), mean corpuscular haemoglobin (MCH), mean corpuscular haemoglobin concentration (MCHC), PLT count, mean PLT volume (MPV) and plateletcrit (PCT).

### 2.3. Echocardiography Protocol

Echocardiography images were recorded before each series and 30 min after the dive to intercept the usually peak of bubbles [25] using a commercially available instrument (MyLab 5, Esaote SPA, Florence, Italy) with a cardiac probe (2.5–3.5 MHz).

All echocardiograms were performed with each subject lying on his left side and breathing normally: recording time was 20 s and all frames were saved in the hard drive for subsequent analysis.

Bubbles were graded according to the Eftedal and Brubakk (EB) scale as follows [33]:

0—no bubbles;

1—occasional bubbles;

2—at least one bubble per 4 heart cycles;

3—at least one bubble per cycle;

4—continuous bubbling, at the least one bubble cm^2^ in all frames;

5—“whiteout”—impossible to see individual bubbles.

After the analysis of the recorded echocardiography frames, divers were divided into two groups: bubblers (B) Grades 3, 4 and 5 and non-bubblers (NB) Grades 0 and 1.

Subjects that showed Grade 2 were excluded by the protocol to create a distance between B and NB.

Differences in dive profile and decompression stress between B and NB were investigated to intercept possible risk factors associated with susceptibility to bubbles developed after dive.

Statistical analysis of the differences in anthropometrical data, age, gender and clinical data was not performed, because the sample was too small.

### 2.4. Statistical Analysis

Data are presented as the mean ± standard deviation (SD) for parametric data and median and range for non-parametric data. Taking the pre diving value as 100% the percentage of changes were calculated in each measurement foreseen by the protocol. They were analyzed after the D’Agostino and Pearson normality test to assume a Gaussian distribution.

Differences between NB and B were investigated by means of one sample t-test for parametric data and the Mann–Whitney U-test for non-parametric data.

A probability lower than 5% was assumed as the threshold to reject the null hypothesis (*p* < 0.05).

## 3. Results

A total of 32 experienced SCUBA divers, 26 males and 6 females, mean age 45.1 ± 12,4; mean height 173.4 cm ± 7.9; mean weight 78.5 kg ± 14.2, and BMI 26.0 ± 3.8 were studied during a single dive in the Y-40 “The Deep Joy” swimming pool (Table 1). The dive profile implied a mean depth of 41.5 ± 0.5 m, a mean diving time of 50.4 ± 7.7 min, mean temperature of 32.7 ± 1.2 °C and a mean GF of 0.83 ± 0.03 (Table 1).

A point-by-point analysis of diving profiles confirmed that all divers respected the diving planning and also the calculated GF at the end of dives confirmed a similar hyperbaric exposure (0.83 ± 0.03).

The echocardiography protocol allowed to allocate 9 B and 19 NB divers while 4 subjects showed intermediate conditions (Grade Two) and were excluded from the protocol.

**Figure 1:**
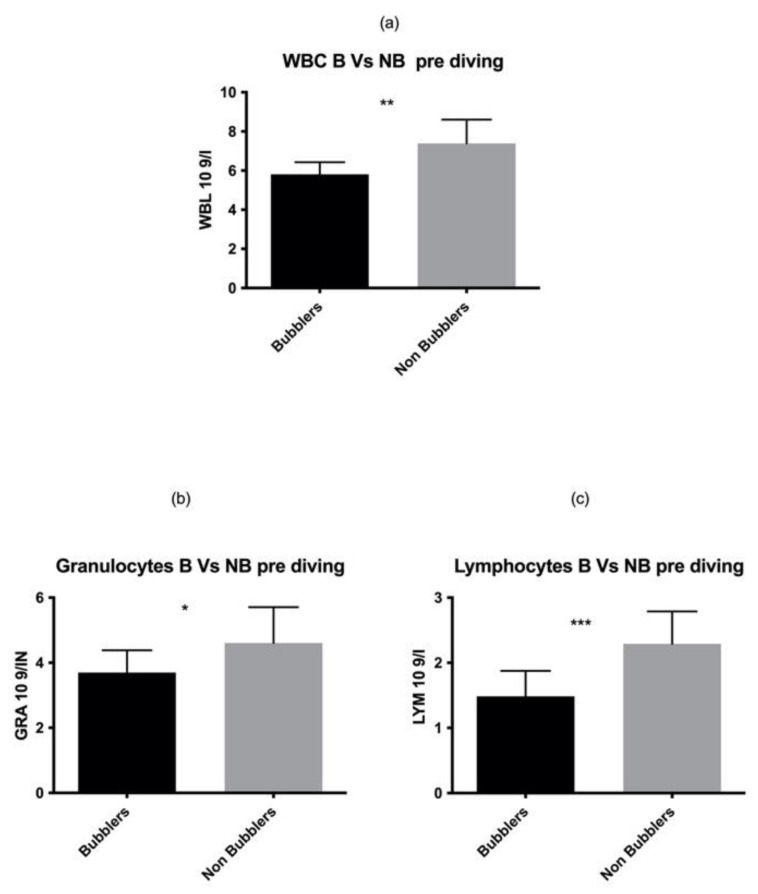
We found a statistically significant higher number of total WBC (**a**) GRAN (**b**) LYM (**c**) in NB as compared to the B divers in the pre diving sample. Asterisks indicate a value significantly different compared to basal (* *p* < 0.05, ** *p* < 0.01, *** *p* < 0.001).

As reported in Figure 1, we found a statistically significant higher number of total WBC in NB as compared to the B divers in the pre diving sample (*p* = 0.001). These data are confirmed by two different elements of white series, GRAN *p* = 0.03, LYM *p* = 0.0003 while no statistical differences were found in MONO *p* = 0.06.

**Figure 2:**
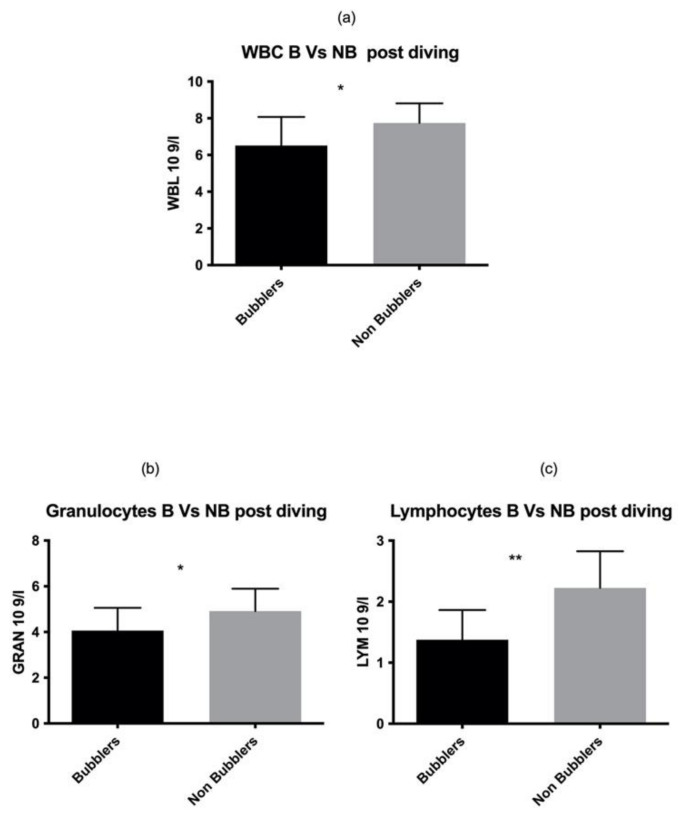
Same results were found also in post diving sample. Asterisks indicate a value significantly different compared to basal (* *p* < 0.05, ** *p* < 0.01).

These data were also confirmed in the post diving blood sample analysis (Figure 2) for both GRAN and LYM (GRAN *p* = 0.02, LYM *p* = 0.001 respectively) (Table 2).

We found statistically significant increase in GRAN (*p* < 0.001) in post diving value with respect to basal value while LYM and MONO did not show any statistical difference (*p* > 0.05) (Table 2).

We did not find any post diving vs. pre diving statistical difference between NB and B for RBC, HCT, MCH and MCHC, while HGB and MCV showed a statistically significant increase after diving (HGB *p* = 0.026, MCV *p* = 0.019). (Table 3).

Finally, PLT and MPV showed a statistically significant increase post diving as compared to pre diving (PLT *p* = 0.01; MPV *p* = 0.0003). Regarding PLT related parameters, we did not find any statistically significant difference in unit per microliter of blood, in mean MPV and PCT, in NB vs. B both in pre diving and post diving values (Table 4).

## 4. Discussion

This study aimed to investigate a possible relation between the evidence of inert gas bubbles and hematological parameters after a SCUBA dive. As a secondary scope, we investigated changes, in the same hematological parameters, between pre and post dive samples. SCUBA diving exposes the human body to a combination of several factors including hyperbaria, hyperoxia, breathing resistance, physical effort and inert gas bubble generation [1]. Bubbles can occur in blood circulation, activating vascular endothelium and inducing inflammation with the activation of leukocytes associated to the production of cytokines [29,34]. After a dive at −42 m for 40 min, we observed an increase in WBC, (GRAN and LYM) according to the data obtained by other authors [5,15,35] even if the protocol was different as time points. Neutrophil mobilization is exacerbated by the production of stress biomarkers such as catecholamines and cortisol [6,36]. All the investigated divers performed similar diving profile and dived into an approximate GF interval, ranged from 0.80 to 90, usually associated to the peak of bubble formation [25], guaranteeing a suitable diving exposure for our purpose.

The most interesting result obtained during our protocol emerged when divers were divided according to their circulating bubble production, into B and NB.

In the NB group, we found a higher amount of WBC (GRAN and LYM) as compared to the B group, already in pre-diving blood sample (therefore not diving related), confirmed in the post dive blood analyses. This aspect could be very important to explain one of the possible causes of inter-subject differences in bubble formation in divers performing the same dive profile [25]. Despite the absence of an investigation related to anti-inflammatory mediator changes, these data may be explained considering that SCUBA-related acute inflammation is modulated by circulating WBC and leads to the stimulation of toll-like receptors (TLRs) and, consequently, the activation of NF-κB pathway [37]. Even if the role of anti-inflammatory mediators were not directly investigated in this protocol, it is known that some authors evaluated the inflammatory status measuring IL-6, IL-8, MIP-1β and other pro-inflammatory molecules [38], including chemokines in recreational divers (Spisni, Marabotti et al. 2017). Some circulating chemokines, namely CCL2 and CCL5, increased after diving: CC chemokines are stored in and released from PLT and activated LYM [39]. Circulating chemokine CCL5 is known to contribute to endothelial activation and the interaction between endothelial cells and MONO [40]. CCL5 secretion facilitates endothelial progenitor cell recruitment and increases NO production in endothelial cells [41], protecting vascular endothelium from endothelial dysfunction. In addition, it is well known that SCUBA diving induces cytokine response [42] and some authors observed the increase of circulating IL-6 IL-8, IL-10, and IL-1b cytokines with anti-inflammatory role [23]. This cytokine cascade seems to be involved in mediating the health beneficial effects of exercise and to play an important role in the modulation of low-grade inflammation induced by exercise oxidative stress [43]. All these mediators are produced by the immune system LYM and GRAN and may explain our results suggesting a protective role of WBC toward the production of bubbles through a WBC mediated anti-inflammatory response in absence of DCS sintom onset. Accordig to our findings and secondary scope, Spisni et al. found an increase in WBC in divers without any episode of DCS [39]. Bubbles in the vascular system can obstruct blood flow activating inflammatory pathways [44]: the WBC increase against inflammation, caused by the bubbles, raises the WBC availability and this could be an additional protective factor.

Increased pO_2_ due to hyperbaric conditions can raise the activity of LYM antioxidant enzymes that may be in part responsible for the reduced levels of H_2_O_2_ during SCUBA diving [3]. In addition, we cannot exclude an active role of neutrophils to remove inert gas (nitrogen) bubbles from the blood by phagocytosis or simple inclusion by diffusion.

We did not find significant changes in RBC and their related factors after diving vs. pre diving and also in their number per cubic ml in NB vs. B. Despite RBC vulnerability to ROS/RNS attack during hyperbaric hyperoxia, endogenous antioxidant defenses were activated to protect RBC from free radical damage. The unchanged RBC value may reflect an unchanged marker of cellular damage in erythrocytes indicating that the cellular antioxidant response is enough to avoid the ROS-induced damage [44].

The HGB and MCV reduction may be explained by the haemolysis that might occur during hyperbaric exposure [45,46].

Circulating bubbles can have inflammatory effects [47] and PLT activation plays a key role in the response to inflammation [48], because their receptors participate in neutrophil extracellular bacteria trapping in septic blood that induces PLT to adhere to neutrophils [49] and also knowing that bubbles cause PLT adhesion in animals [50,51] and humans reducing free circulating PLT [52]. On the others hand, PLT changes during SCUBA diving are controversial: some authors found a decrease of PLT count related to bubble formation [32,53] that may be due to individual physiology, dive profile and diver training/experience. PLT activation by bubbles is a slow process that involves several changes of plasma proteins at the bubble interface. After dive, lungs eject many inert gas bubbles minimizing PLT activation related inflammation and thrombosis. Before this process is complete, many bubbles can disappear and this delay probably may contribute to the latency period of DCS [54]. Some authors observed that breathing hyperbaric or normobaric O_2_ before a dive reduced PLT activation as consequence of O_2_ increase in fat tissue, reducing decompression-induced air bubble formation and alleviating decompression induced PLT activation [55]. On the other hand, increased pO_2_, breathing nitrox mixtures, can reduce the level of decompression induced PLT activation [56]. The small numbers of subject investigated does not permit to give firm conclusion about the relationship between PLT count and bubble formation.

We did not find relationship between PLT count, PLT-related parameters and bubble formation. On the other hand, we found a statistically significant post dive increase in PLT count and MPV.

However, no differences were found in PLT levels and their associated parameters between B and NB.

The main limitation of this study is only one blood sample, carried out 30 min after the surfacing, is not enough to evaluate the WBC kinetics that could be a relevant information in future study in this field. Another limitation is the absence of investigation about cytokine and inflammatory marker trends.

Finally, the imbalance between the number of male and female divers and the reduced sample size in this study represent a limitation according to our point of view.

## 5. Conclusions

We found a higher number of WBC (GRAN and LYM) in NB, both in pre and post diving value, as compared to B that could explain a different predisposition to bubble formation. Even if the role of anti-inflammatory mediators were not directly investigated in this protocol, these data may be explained considering that SCUBA-related acute inflammation is modulated by circulating WBC. No interesting differences were found concerning RBC and PLT between B and NB. Further experiments will be necessary to understand better our results, raising the number of female divers and investigating the circulating cytokines. If confirmed in future similar protocols with a larger number of subjects, our result may represent a real support for the SCUBA diving community allowing a prediction of the subjects with the higher possibility to develop bubbles in bloodstream after SCUBA diving.

## Figures and Tables

**Table 1:** Anthropometric data and dive parameters.

Anthropometric Data	Means ± Ds	B vs. NB
Age	45.0 ± 11.8	N.S. ^1^
Height	173.4 ± 7.9	N.S.
Weight	79.7 ± 14.6	N.S.
BMI	26.4 ± 4.1	N.S.
**Dive Parameters**		
Maximum depth	41.5 ± 0.5	N.S.
Diving time	50.4 ± 7.7	N.S.
Temperature	32.7 ± 1.2	N.S.
Maximum GF at the end	0.83 ± 0.03	N.S.

^1^ Not statistically significant.

**Table 2:** WBC and WBC-related parameters.

White Series	B	NB	Results
WBC pre (10^9^)	5.81 ± 0.62	7.39 ± 1.21	*p* = 0.001
WBC post (10^9^)	6.51 ± 1.56	7.74 ± 1.07	*p* = 0.021
GRANs pre (10^9^)	3.70 ± 0.69	4.61 ± 1.01	*p* = 0.031
GRAN post (10^9^)	4.07 ± 0.99	4.92 ± 0.98	*p* = 0.041
LYM pre (10^9^)	1.49 ± 0.39	2.29 ± 0.50	*p* = 0.0003
LYM post (10^9^)	1.38 ± 0.49	2.23 ± 0.60	*p* = 0.001
MONO pre (10^9^)	0.39 ± 0.10	0.49 ± 0.12	N.S. ^1^
MONO post (10^9^)	0.42 ± 0.13	0.42 ± 0.11	N.S.
	Pre	Post	
WBC (10^9^)	6.82 ± 1.23	7.32 ± 1.34	*p* = 0.0050
GRAN (10^9^)	4.19 ± 1.06	4.60 ± 1.00	*p* = 0.001
LYM (10^9^)	2.09 ± 0.62	2.00 ± 0.71	N.S.
MONO (10^9^)	0.45 ± 0.13	0.43 ± 0.11	N.S.

^1^ Not statistically significant.

**Table 3:** RBC and RBC related parameters.

RBC	B	NB	Results
RBC pre (10^12^)	5.13 ± 0.79	3.92 ± 0.95	N.S. ^1^
RBC post (10^12^)	5.26 ± 0.56	5.20 ± 0.65	N.S.
HGB pre (g/L)	15.94 ± 1.60	15.02 ± 2.45	N.S.
HGB post (g/L)	15.74 ± 1.02	14.66 ± 1.05	N.S.
MCV pre (fL)	86.36 ± 3.85	85.49 ± 13.36	N.S.
MCV post (fL)	86.97 ± 3.90	83.78 ± 9.72	N.S.
HCT pre (%)	45.09 ± 2.82	42.88 ± 5.81	N.S.
HCT post (%)	45.48 ± 3.13	43.03 ± 3.75	N.S.
MCH pre (pg)	30.56 ± 1.90	28.83 ± 4.61	N.S.
MCH post (pg)	30.12 ± 2.34	28.47 ± 4.20	N.S.
MCHC pre (g/L)	35.14 ± 2.02	33.99 ± 2.20	N.S.
MCHC post (g/L)	34.64 ± 1.50	33.38 ± 3.22	N.S.
	Pre	Post	
RBC(10^12^)	5.12 ± 0.88	5.22 ± 0.61	N.S.
HGB (g/L)	15.32 ± 2.23	15.01 ±1.84	*p* = 0.026
MCV (fL)	85.77 ± 11.11	84.77 ± 8.35	*p* = 0.019
HCT (%)	43.59 ± 5.10	43.81 ±3.69	N.S.
MCH (pg)	29.38 ± 3.99	29.00 ± 3.74	N.S.
MCHC pre (g/L)	34.36 ± 2.17	33.79 ± 2.82	N.S.

^1^ Not statistically significant.

**Table 4:** Platelets and platelets related parameters.

Platelet	B	NB	Results
PLT pre (10^9^)	183.20 ± 56.3	196.80 ± 70.9	N.S. ^1^
PLT post (10^9^)	198.8 ± 55.4	218.2 ± 67.6	N.S.
MPV pre (fL)	9.56 ± 0.92	9.29 ± 1.03	N.S.
MPV pre (fL)	9.20 ± 1.26	8.67 ± 1.17	N.S.
PCT pre (%)	0.17 ± 0.06	1.01 ± 3.63	N.S.
PCT post (%)	0.18 ± 0.05	0.19 ± 0.05	N.S.
	Pre	Post	
PLT (10^9^)	192.5 ± 65.89	212.0 ± 63.56	*p* = 0.01
MPV (fL)	12.56 ± 16.97	8.85 ± 1.20	*p* = 0.0003
PCT (%)	0.74 ± 2.99	0.18 ± 0.05	N.S.

^1^ Not statistically significant.

## Data Availability

Original datasets are available upon request after anonymization of personal data.
